# Supplementary material for: Heat adaptation of phage T7 under an extended genetic code
Source: Virus Evol. 2021 Dec 1;7(2):veab100. doi: 10.1093/ve/veab100 (PMC8923235; doi:10.1093/ve/veab100)
Supplement: veab100_Supp [file veab100_supp.zip › Sequencing_Analysis.zip!/Sequencing_Analysis/S3/evidence/JC_SIDE_2_44.html]

BRESEQ :: Evidence


| New junction evidence | | | | | | | | | | | |
| --- | --- | --- | --- | --- | --- | --- | --- | --- | --- | --- | --- |
|  | | seq id | position | reads (cov) | reads (cov) | score | skew | freq | annotation | gene | product |
| \* | ? | NC\_001604.1 | = 281 | 1619 (1.060) | 375 (0.230) +TAAAGACCAGACC | 16/508 | 34.0 | 17.7% | intergenic (–/–) | *–/–* | –/– |
|  | ? | NC\_001604.1 | 282 = | 1822 (1.060) |  |  |  |  | intergenic (–/–) | *–/–* | –/– |
| Rejected: Coverage evenness skew score above cutoff. | | | | | | | | | | | |
| Rejected: Frequency below/above cutoff threshold. | | | | | | | | | | | |

Only 100 of 2198 total aligned reads displayed.  

`‑‑‑‑‑‑‑‑‑‑‑‑TCTCACAGTGTACGGACCTAAAGTTCCCCCATAGGGGGTACCTAAAG‑CCCAGCCAATCACCTAAAGTCAACCTTCGGTTGACCTTGAGGGTTCCCTAAGGGTT‑GGGGATGACCCTTGGGTTTGTCTTTGGGTGTTACCTTGA‑‑GTGTCTCTCTGTGTCCCTATCTGTTACAGTCTCCTAAAGTATCCTCCTAAAGTCACCTCCTAACGTCCATCCTAAAGCCAACACCTAAAGCCTACACCTAAAGACCCATCAAGTCAACGCCTATCTTAAAGTTTAAACATAAAGACCAGACCTAAAGACCAGACCTAAAGACACTACATAAAGACCAGACCTAAAGACGCCTTGTTGTTAGCCATAAAGTGATAACCTTTAATCATTGTCTTTATTAATACAACTCACTATAAGGAGAGACAACTTAAAGAGACTTAAAAGATTAATTTAAAATTTATCAAAAAGAGTATTGACTTAAAGTCTAACCTATAGGATACTTACAGCCATCGAGAGGGACACGGCGAATAGCCATCCCAATCGACACCGGGGTCAACCGGATAAGTAGACAGCCTGATAAGTCGCACGAAAAA  >  NC_001604.1/1‑582`  

                                                                                                                                                                                                                                                                                                         `|`                                                                                                                                                                                                                                                                                                              
`agatgtgtataagagacaggtggACGGACCTAAAGTTCCCCCAGAGGGGGTACCTAAAG‑CCCAGCCAATCACCTAAAGTCAACCTTCGGTTGACCTTGAGGGTTCCCTAAGGGTT‑GGGGATGACCCTTGGGTTTGTCTTTGGGTGTTACCTTGA‑‑GTGTCTCTCTGTGTCCCTATCTGTTACAGTCTCCTAAAGTATCCTCCTAAAGTCACCTCCTAACGTCCATCCTAAGGCCAACACCTAAAGCCTACACCTAAAGACCCATCAAGTCAACGCCTATCTTAAAGTTTAAACAtaaagacc                                                                                                                                                                                                                                                                                                       <  1:260085/282‑1 (MQ=255)`  
         `atgtgtataagagacaggACCTAAAGTTCCCCCATAGGGGGTACCTAAAG‑CCCAGCCAATCACCTAAAGTCAACCTTCGGTTGACCTTGAGGGTTCCCTAAGGGTT‑GGGGATGACCCTTGGGTTTGTCTTTGGGTGTTACCTTGA‑‑GTGTCTCTCTGTGTCCCTATCTGTTACAGACTCCTAAAGTATCCTCCTAAAGTCACCTCCTAACGTCCATCCTAAAGCCAACACCTAAAGCCTACACCTAAAGACCCATCAAGTCAACGCCTATCTTAAAG‑TTAAACAtaaagaccagacctaaag                                                                                                                                                                                                                                                                                             <  1:150453/285‑1 (MQ=255)`  
          `aagagacagggtaaCGGACCTAAAGTTCCCCCATAGGGGGTACCTAAAG‑CCCAGCCAATCACCTAAAGTCAACCTTCGGTTGACCTTGAGGGTTCCCTAAGGGTT‑GGGGATGACCCTTGGGTTTGTCTTTGGGTGTTACCTTGA‑‑GTGTCTCTCTGTGTCCCTATCTGTTACAGTCTCCTAAAGTATCCTCCTAAAGTCACCTCCTAACGTCCATCCTAAAGCCAACACCTAAAGCCTACACCTAAAGACCCATCAAGTCAACGCCTATCTTAAAGTTTAAACAtaaagaccagacctaaag                                                                                                                                                                                                                                                                                             <  1:71785/288‑1 (MQ=255)`  
            `gagacaggtgtACGGACCTAAAGTTCCACCATAGGGGGTACCTAAAG‑CCCAGCCAATCACCTAAAGTCAACCTTCGGTTGACCTTGAGGGTTCCATAAGGGTT‑GGGGATGACCCTTGGGTTTGTCTTTGGGTGTTACCTTGA‑‑GTGTCTCTCTGTGTCCCTAGCTGTGACAGTCTCCTAAAGTATCCTCCTAAAGTCACCTCCTAACGTCCATCCTAAAGCCAACACCTAAAGCCTACACCTAAAGACCCATCAAGTCAACGCCTATCTTAAAGTTTAAACAtaaagaccagacctaaagac                                                                                                                                                                                                                                                                                           <  1:222184/294‑1 (MQ=255)`  
                   `gtgtACGGACCTAAAGTTCCCCCATAGGGGGTACCTAAAG‑CCCAGCCAATCACCTAAAGTCAACCTTCGGTTGACCTTGAGGGTTCCCTAAGGGTT‑GGGGATGACCCTTGGGTTTGTCTTTGGGTGTTACCTTGA‑‑GTGTCTCTCTGTGTCCCTATCTGTTACAGTCTCCTAAAGTATCCTCCTAAAGTCACCTCCTAACGTCCATCCTAAAGCCAACACCTAAAGCCTACACCTAAAGACCCATCAAGTCAACGCCTATCTTAAAGTTTACACATaaagaccagacctaaagaccagacct                                                                                                                                                                                                                                                                                    >  1:9078/1‑301 (MQ=255)`  
                   `gtgtACGGACCTAAAGTTCCCCCATAGGGGGTACCTAAAG‑CCCAGCCAATCACCTAAAGTCAACCTTCGGTTGACCTTGAGGGTTCCCTAAGGGTT‑GGGGATGACCCTTGGGTTTGTCTTTGGGTGTTACCTTGA‑‑GTGTCTCTCTGTGTCCCTATCTGTTACAGTCTCCTAAAGTATCCTCCTAAAGTCACCTCCTAACGTCCATCCTAAAGCCAACACCTAAAGCCTACACCTAAAGACCCATCAAGTCAACGCCTATCTTAAAGTTTAAACAtaaagaccagacctaaagaccctgtct                                                                                                                                                                                                                                                                                    >  1:214155/1‑295 (MQ=255)`  
                   `gtgtACGGACCTAAAGTTCCCCCATAGGGGGTACCTAAAG‑CCCAGCCAATCACCTAAAGTCAACCTTCGGTTGACCTTGAGGGTTCCCTAAGGGTT‑GGGGATGACCCTTGGGTTTGTCTTTGGGTGTTACCTTGA‑‑GTGTCTCTCTGTGTCCCTATCTGTTACAGTCTCCTAAAGTATCCTCCTAAAGTCACCTCCTAACGTCCATCCTAAAGCCAACACCTAAAGCCTACACCTAAAGACCCATCAAGTCAACGCCTATCTTAAAGTTTAAACATaaagaccagacctaaagacaagacct                                                                                                                                                                                                                                                                                    >  1:131025/1‑301 (MQ=255)`  
                   `gtgtACGGACCTAAAGTTCCCCCATAGGGGGTACCTAAAG‑CCCAGCCAATCACCTAAAGTCAACCTTCGGTTGAACCAGAGGGTTCCCTAAGGGTT‑GGGGATGACCCTTGAGTTTGTCTTTGGGTGTTACCTTGA‑‑GTGTCTCTCTGTGTCCCTATCTGTTAAAGTCTCCTAAAGCATCCTCCTACAGTCACCTCCTAACGTCCATCCTAAAGCCAACACCTAAAGCCTACACCTAAAGACCCATCAAGTCAACGCCTATCTTAAAGTTCAAACAtaaagacaagacctaaagaacagacat                                                                                                                                                                                                                                                                                    >  1:340222/1‑299 (MQ=25)`  
                   `gtgtACGGACCTAAAGTTCCCCCATAGGGGGTACCTAAAG‑CCCAGCCAATCACATAAAGTCAACCTTCGGTTGACCTTGAGGGTTCCCTAAGGGTT‑GGGGATGACCCTTGGGTTTGTCTTTGGGTGTTACCTTGA‑‑GTGTCTCTCTGTGTCCCTATCTGTTACAGTCTCCTAAAGTATCCTCCTAAAGTCACCTCCTAACGTCCATCCTAAAGCCAACACCTAAAGCCTACACCTAAAGACCCATCAAGTCAACGCCTATCTTAAAGTTTAAACATaaagaccagacctaaagaccagacct                                                                                                                                                                                                                                                                                    >  1:3493/1‑301 (MQ=255)`  
                     `gtACGGACCTAAAGTTCCCCCATAGGGGGTACCTAAAG‑CCCAGCCAATCACCTAAAGTCAACCTTCGGTTGACCTTGAGGGTTCCCTAAGGGTT‑GGGGATGACCCTTGGGTTTGTCTTTGGGTGTTACCTTGA‑‑GTGTCTCTCTGTGTCCCTATCTGTTACAGTCTCCTAAAGTATCCTCCTAAAGTCACCTCCTAACGTCCATCCTAAAGCCAACACCTAAAGCCTACACCTAAAGACCCATCAAGTCAACGCCTATCTTCAAGTTTAAACAtaaagaccagacctaaagctgtcccttaa                                                                                                                                                                                                                                                                                  >  1:28762/1‑290 (MQ=255)`  
                     `gtACGGACCTAAAGTTCCCCCATAGGGGGTACCTAAAG‑CCCAGCCAATCACCTAAAGTCAACCTTCGGTTGACCTTGAGGGTTCCCTAAGGGTT‑GGGGATGACCCTTGGGTTTGTCTTTGGGTGTTACCTTGA‑‑GTGTCTCTCTGTGTCCCTATCTGTTACAGTCTCCTAAAGTATCCTCCTAAAGTCACCTCCTAACGTCCATCCTAAAGCCAACACCTAAAGCCTACACCTAAAGACCCATCAAGTCAACGCCTATCTTAAAGTTTAAACATAAagaccagacctacagaccagacctaa                                                                                                                                                                                                                                                                                  >  1:313805/1‑301 (MQ=255)`  
                     `gtACGGACCTAAAGTTCCCCCATAGGGGGTACCTAAAG‑CCCAGCCAATCACCTAAAGTCAACCTTCGGTTGACCTTGAGGGTTCCCTAAGGGTT‑GGGGATGACCCTTGGGTTTGTCTTTGGGTGTTACCTTGA‑‑GTGTCTCTCTGTGTCCCTATCTGTTACAGTCTCCTAAAGTATCCTCCTAAAGTCACCTCCTAACGTCCATCCTAAAGCCAACACCTAAAGCCTACACCTAAAGACCCATCAAGTCAACGCCTATCTTAAAGTTTAAACATAAagaccagacctaaagaccagacctaa                                                                                                                                                                                                                                                                                  >  1:272962/1‑301 (MQ=255)`  
                          `gACCTAAAGTTCCCCCATAGGGGGTACCTAAAG‑CCCAGCCAATCACCTAAAGTCAACCTTCGGGTGACCTTGAGGGTTCCCTACGGGTT‑GGGGATGACCCTTTGGTTTGTCTTTGGGTGTTACCTTGA‑‑GTGTCTCTTTGTGTCACTATCTGTAACAGTCTCCTAAAGTATCCTCCTAAAGTCACCTCCTAACGTCCATCCTAAAGCCAACACCTAAAGCCTACACCTAAAGATCCATCAAGTCAACGCCTTTCTTAAAGTTTAAACTTAAATACCAGACCTAAACACAAGACCTAAAGcca                                                                                                                                                                                                                                                                             >  1:200702/1‑301 (MQ=25)`  
                           `aCCTAAAGTTCCCCCATAGGGGGTACCTAAAG‑CCCAGCCAATCACCTAAAGTCAACCTTCGGTTGACCTTGAGGGTTCCCTAAGGGTT‑GGGGATGACCCTTGGGTTTGTCTTTGGGTGTTACCTTGA‑‑GTGTCTCTCTGTGTCCCTATCTGTTACAGTCTCCTAAAGTATCCTCCTAAAGTCACCTCCTAACGTCCATCCTAAAGCCAACACCTAAAGCCTACACCTAAAGACCCATCAAGTCAACGCCTATCTTAAAGTTTAAACATAAAGACCAGACCTAAAGACCAGACCTAAAGacac                                                                                                                                                                                                                                                                            <  1:205303/301‑1 (MQ=255)`  
                            `ccTAAAGTTCCCCCATAGGGGGTACCTAAAG‑CCCAGCCAATCACCTAAAGTCAACCTTCGGTTGACCTTGAGGGTTCCCTAATGGTT‑GGGGATGACCCTTGGGTTTGTCTTTGGGTGTTACCTTGA‑‑GTGTCTCTCTGTGTCCCTATCTGTTACAGTCTCCTAAAGTATCCTCCTAAAGTAACCTCCTAACGTCCATCCTAAAGCCAACACCTAAAGCCTACACCTAAAGACCCATCAAGTCAACGCCTATCTTAAAGTTTAAACATAAAGACCAGACCTAAAGCCCAGACCTAAAGacacc                                                                                                                                                                                                                                                                           >  1:180428/1‑300 (MQ=255)`  
                                `gtataagagacaggggggTACCTAAAG‑CCCAGCCAATCACCTAAAGTCAACCTTCGGTTGACCTTGAGGGTTCCCTAAGGGTT‑GGGGATGACCCTTGGGTTTGTCTTTGGGTGTTACCTTGA‑‑GTGTCTCTCTGTGTCCCTATCTGTTACAGTCTCCTAAAGTATCCTCCTAAAGTCACCTCCTAACGTCCATCCTAAAGCCAACACCTAAAGCCTACACCTAAAGACCCATCAAGTCAACGCCTATCTTAAAGTTTAAACATAAAGACCAGACCTAAAGACCAGACCTAAAGACACTACAt                                                                                                                                                                                                                                                                       <  1:97329/288‑1 (MQ=255)`  
                                     `cccccATAGGGGGTACCTAAAG‑CCCAGCCAATCACCTAAAGTCAACCTTCGGTTGACCTTGAGGGTTCCCTAAGGGTT‑GGGGATGACCCTTGGGTTTGTCTTTTGGTGTTACCTTGA‑‑GTGTCTCTCTGTGTCCCTATCTGTTACAGTCTCCTAAAGTATCCTCCAAAAGTCACCTCCTAACGTCCATCCTCAAGCCAACACCTAAAGCCTACACCAAAAGCCCCATCAAGTCACCGCCTATCTTAAATTTTAAACATAAAGCCcagaactcaagaccaaacctaaataccccctgtccctt                                                                                                                                                                                                                                                                  >  1:163801/1‑289 (MQ=25)`  
                                     `cccccATAGGGGGTACCTAAAG‑CCCAGCCAATCACCTAAAGTCAACCTTCGGTTGACCTTGAGGGTTCCCTAAGGGTT‑GGGGATGACCCTTGGGTTTGTCTTTGGGTGTTACCTTGA‑‑GTGTCTCTCTGTGTCCCTATCTGTTACAGTCTCCTAAAGTATCCTCCTAAAGTCACCTCCTAACGTCCATCCTAAAGCCAACACCTAAAGCCTACACCTAAAGACCCATCAAGTCAACGCCTATCTTAAAGTTTAAACATAAAGACCAGACCTAAAGACCAGACCTAAAGACACTACATAAaga                                                                                                                                                                                                                                                                  <  1:127748/301‑1 (MQ=255)`  
                                      `ccccATAGGGGGTAACTAAAG‑CCCAGCAAATCCCCTAAAGTCAACCGTCGGTTGACCTTGAGGGTTCCCTAAGGGTT‑GGGGATGACCCTTGGGTTTGTCTTTGGGTGGTACCTTGA‑‑GTGTCTCTCTGTGTCCCTATCTGTTACAGTCTCCTAAAGTATCCGCCTAAAGTCACCTCCTAACGTCCATCCTAAAGCCAACACCTAAAGCCTACACCTAAAGACCCATCAAGTCAACGCCTATCTTAAAGTTTAAAAATAAAGACCAGACCTAAAGACCAGACCTAAAGACACTACATAAagac                                                                                                                                                                                                                                                                 <  1:154115/301‑1 (MQ=255)`  
                                       `cccATAGGGGGTACCTAAAG‑CCCAGCCAATCACCTAAAGTCAACCTTCGGTTGACCTTGAGGGTTCCCTAAGGGTT‑GGGGATGACCCTTGGGTTTGTCTTTGGGTGTTACCTTGA‑‑GTGTCTCTCTGTGTCCCTATCTGTTACAGTCTCCTAAAGTATCCTCCTAAAGTCACCTCCTAACGTCCATCCTAAAGCCAACACCTAAAGCCTACACCTAAAGACCCATCAAGTCAACGCCTATCTTAAAGTTTAAACATAAAGACCAGACCTAAAGACCAGACCTAAAGACACTACATAAagacc                                                                                                                                                                                                                                                                >  1:301855/1‑301 (MQ=255)`  
                                       `cccATAGGGGGTACCTAAAG‑CCCAGCCAATCACCTAAAGTCAACCTTCGGTTGACCTTGAGGGTTCCCTAAGGGTT‑GGGGATGACCCTTGGGTTTGTCTTTGGGTGTTACCTTGA‑‑GTGTCTCTCTGTGTCCCGATCTATTACAGTCTCCTAAAGTATCCTCCTAAAGTCACCTCCTAACGTCCATCCTAAAGCCAACACCTAAAGCCTACACCTAAAGACCCATCAAGTCAACGCCTATCTTAAAGTTTAAACATAAAGACcagacctaaagaccagacctaaagacctgtctcttataca                                                                                                                                                                                                                                                                >  1:143286/1‑287 (MQ=255)`  
                                        `ccATAGGGGGTACCTAAAG‑CCCAGCCAATCACCTAAAGTCAACCTTCGGTTGACCTTGAGGGTTCCCTAAGGGTT‑GGGGATGACCCTTGGGTTTGTCTTTGGGTGTTACCTTGA‑‑GTGTCTCTCTGTGTCCCTATCTGTTACAGTCTCCTAAAGTATCCTCCTAAAGTCACCTCCTAACGTCCATCCTAAAGCCAACACCTAAAGCCTACACCTAAAGACCCATCAAGTCAACGCCTATCTTAAAGTTTAAACATAAAGACCAGACCTAAAGACCAGACCTAAAGACACTACATAAagacca                                                                                                                                                                                                                                                               >  1:281733/1‑301 (MQ=255)`  
                                            `aGGGGGTAACTAAAGCCCCTGCCAACCA‑CTAAAGTCAACCTTCGGTTGACCTTGAGGGTTCCCTAAGGGTT‑GGGGATGACCCTTGGGTTTGTCTTTGGGTGTTACCTTGA‑‑GTGTCTCTCTGTGTCCCTATCTGTTACAGTCTCCTAAAGTAACCTCCGAAAGTCACCTCCTAACGTCCATCCTAAAGCCAACACCTAAAGCCTACACCTAAAGACCCATCAAGTCAACGCCTATCTTAAAGTTTAAACATAAAGACCAGACCTAAAGACCAGACCTAAAGACACTACATAAagaccagacc                                                                                                                                                                                                                                                           <  1:265678/301‑1 (MQ=255)`  
                                                   `aCCTAAAG‑CCCAGCCAATCACCTAAAGTCAACCTTCGGTTGACCTTGAGGGTTCCCTAAGGGTT‑GGGGATGACCCTTGGGTTTGTCTTTGGGTGTTACCTTGA‑‑GTGTCTCTCTGTGTCCCTATCTGTTACAGTCTCCTAAAGTATCCTCCTAAAGTCACCTCCTAACGTCCATCCTAAAGCCAACACCTAAAGCCTACACCTAAAGACCCATCAAGTCAACGCCTCTCTTAAAGTTTAAACATAAAGACCAGACCTAAAGACCAGACCTAAAGACACTACATAAAGCCCAGACCTAAAgcc                                                                                                                                                                                                                                                    >  1:168557/1‑299 (MQ=255)`  
                                                    `ccTAAAG‑CCCAGCCAATCACCTAAAGTCAACCTTCGGTTGACCTTGAGGGTTCCCTAAGGGTT‑GGGGATGACCCTTGGGTTTGTCTTTGGGTGTTACCTTGA‑‑GTGTCTCTCTGTGTCCCTATCTGTTACAGTCTCCTCAAGTATCCTCCTAAAGTCACCTCCTACCGTCCATCCTAAAGCCAACACCTAAAGCCTACACCTAAAGACCCATCAAGTCAACGCCTATCTTAAAGTTTAAACATAAAGACCAGACCTAAAGACCAGACCTAAAGACACTACATAAagaccctgtctcttatac                                                                                                                                                                                                                                                   >  1:147840/1‑288 (MQ=255)`  
                                                                  `cAATCACCTAAAGTCAACCTTCGGTTGACCTTGAGGGTTCCCTAAGGGTT‑GGGGATGACCCTTGGGTTTGTCTTTGGGTGTTACCTTGA‑‑GTGTCTCTCTGTGTCCCTATCTGTTCCAGTCTCCTAAAGTATCCTCCTAAAGTCACCTCCTAACGTCCATCCTAAAGCCAACACCTAAAGCCTACACCTAAAGACCCATCAAGTCAACGCCTATCTTAAAGTTTAAACATAAAGACCAGACCTAAAGACCAGACCTAAAGACACTACATAAAGACCAGACCTAAAGACGCCTTGTTGTTAGc                                                                                                                                                                                                                                      <  1:43526/301‑1 (MQ=255)`  
                                                                  `cAATCACCTAAAGTCAACCTTCGGTTGACCTTGAGGGTTCCCTAAGGGTT‑GGGGATGACCCTTGGGTTTGTCTTTGGGTGTTACCTTGA‑‑GTGTCTCTCTGTGTCCCTATCTGTTACAGTCTCCTAAAGTATCCTCCTAAAGTCACCTCCTAACGTCCATCCTAAAGCCAACACCTAAAGCCTACACCTAAAGACCCATCAAGTCAACGCCTATCTTAAAGTTTAAACATAAAGACCAGACCTAAAGACCAGACCTAAAGACACTACATAAAGACCAGACCTAAAGACGCCTTGTTGTTAGc                                                                                                                                                                                                                                      <  1:275430/301‑1 (MQ=255)`  
                                                                        `ccTAAAGTCAACCTTCGGTTGACCTTGAGGGTTCCCTAAGGGTT‑GGGGATGACCCTTGGGTTTGTCTTTGGGTGTTACCTTTA‑‑GTGTCTCTCTGTGTCCCTATCTGTTACAGTCTCCTAAAGTATCCTCCTAAAGTCACCTCCTAACGTCCATCCTAAAGCCAACACCTAAAGCCTACACCTAAAGACCCATCAAGTCAACGCCTATCTTAAAGTTTAAACATAAAGACCAGACCTAAAGACCAGACATAAAGACACTACATAAAGACCAGACCTAAAgctgtcccttatacacatctccg                                                                                                                                                                                                                                >  1:161239/1‑279 (MQ=255)`  
                                                                        `ccTAAAGTCAACCTTCGGTTGACCTTGAGGGTTCCCTAAGGGTT‑GGGGATGACCCTTGGGTTTGTCTTTGGGTGTTACCTTGA‑‑GTGTCTCTCTGTGTCCCTATCTGTTACAGTCTCCTAAAGTATCCTCCTAAAGTCACCTCCTAACGTCCATCCTAAAGCCAACACCTAAAGCCTACACCTAAAGACCCATCAAGTCAACGCCTATCTTAAAGTTTAAACATAAAGACCAGACCTAAAGACCCGACCTAAAGACACTACATAAAGACCAGACCTAAAGACGCCTCGTTGTTAGCCATaaa                                                                                                                                                                                                                                >  1:254540/1‑301 (MQ=255)`  
                                                                          `tAAAGTCAACCTTCGGTTGACCTTGAGGGTTCCCTAAGGGTT‑GGGGATGACCCTTGGGTTTGTCTTTGGGTGTTACCTTGA‑‑GTGTCTCTCTGTGTCCCTATCTGTTACAGTCTCCTAAAGTATCCTCCTAAAGTCACCTCCTAACGTCCATCCTAAAGCCAACACCTAAAGCCTACACCTAAAGACCCATCAAGTCAACGCCTATCTTAAAGTTTAAACATAAAGACCAGACCTAAAGACCAGACCTAAAGACACTACATAAAGACCAGACCTAAAGACGCCTTGTTGTTAGCCATAAAGt                                                                                                                                                                                                                              <  1:277979/301‑1 (MQ=255)`  
                                                                             `aGTCAACCTTCGGTTGACCTTGAGGGTTCCCTAAGGGTT‑GGGGATGACCCTTGGGTTTGTCTTTGGGTGTTACCTTGAGTGTGTCTCTCTGTGTCCCTATCTGTTACAGTCTCCTAAAGTATCCTCCTAAAGTCACCTCCTAACGTCCATCCTAAAGCCAACACCTAAAGCCTACACCTAAAGACCCATCAAGTCAACGCCTATCTTAAAGTTTAAACATAAAGACCAGACCTAAAGACCAGACCTAAAGACACTACATAAAGACCAGACCTAAAGACGCCTTGTTGTTAGCCATAAAGTg                                                                                                                                                                                                                             >  1:337191/1‑301 (MQ=255)`  
                                                                                 `aaCCTTCGGTTGTCCTTGAGGGTTCCCTAAGGGTT‑GGGGATGACCCTTGGGTTTGTCTTTGGGTGTTACCTTGA‑‑GTGTCTCTCTGTGTCCCTATCTGTTACAGTCTCCTAAAGTATCCTCCTAAAGTCACCTCCTAACGTCCATCCTAAAGCCAACACCTAAAGCCTACACCTAAAGACCCATCAAGTCAACGCCTATCTTAAAGTTTAAACATAAAGACCAGACCTAAAGACCAGACCTAAAGACACTACATAAAGACCAGACCTAAAGACGCCTTGTTGTTAGCCATAAAGTGATAAcc                                                                                                                                                                                                                       <  1:3324/301‑1 (MQ=255)`  
                                                                                        `ggTTTACCTTGAGGGTTCCCTAAGGGTT‑TGGGATGACCCTTGGGTTTGTCTTTGGGTGTTACCTTGA‑‑GTGTCTCTCTGTGTCCCTATCTGTTACAGTCTCCTAAAGTATCCTCCTAAAGTCACCTCCTAACGTCCATCCTAAAGCCAACACCTAAAGCCTACACCTAAAGACCCATCAAGTCAACGCCTATCTTAAAGTTTAAACATAAAGACCAGACCTAAAGACCAGACCTAAAGACACTACATAAAGACCAGACCTAAAGACGCCTTGTTGTTAGCCATAAAGTGATAACCTTTAATc                                                                                                                                                                                                                <  1:304647/301‑1 (MQ=255)`  
                                                                                        `ggTTGACCTTGAGGGTTCCCTAAGGGTT‑GGGGACGACCCTTGGGTTTTTCTTTGGGTGTTACCTTGA‑‑GTGTCTCTCTGTGTCCCTATCTGTTACAGTCTCCTAAAGTATCCTCCTAAAGTCACCTCCTAACGTCCATCCTAAAGCCAACACCTAAAGCCTACACCTAAAGACCCATCAAGTCAACGCCTATCTTAAAGTTTAAACATAAAGACCAGACCTAAAGACCAGACCTAAAGACACTACATAAAGACCAGACCTAAAGACGCCTTGTTGTTAGCCATAAAGTGATAACCTTTAATc                                                                                                                                                                                                                <  1:41161/301‑1 (MQ=255)`  
                                                                                        `ggTTGACCTTGAGGGTGCCCTAAGGGTT‑GGGGATGACCCTTGGGTTTGTCTTTGGGTGTTACCTTGA‑‑GTGTCTCTCTGTGTCCCTATCTGTTACAGTCTCCTAAAGTATCCTCCTAAAGTCACCTCCTAACGTCCATCCTAAAGCCAACACCTAAAGCCTACACCTAAAGACCCATCAAGTCAACGCCTATCTTAAAGTTTAAACATAAAGACCAGACCTAAAGACCAGACCTAAAGACACTACATAAAGACCAGACCTAAAGACGCCTTGTTGTTAGCCATAAAGTGATAACCTTTAATc                                                                                                                                                                                                                <  1:324413/301‑1 (MQ=255)`  
                                                                                         `gTTGTCCTTGAGGGTTCCCGAAGGGTTGGGGGATGTCCCTTGTGTTTGTCTTTGGGTGTTACCTTGA‑‑GTGTCTCTCTGTGTCCCTATCTGTTACCGTCTCCTAAAGTAGCCTCCTAAAGTCACCTCCTAACGTCCATCCTAAAGCCAACACCTAAAGCCTACACCTAAAGACCCATCAAGTCAACGCCTATCTTAAAGTTTAAACATAAAGACCAGACCTAAAGACCAGACCTAAAGACACTACATAAAGACCAGACCTAAAGACGCCTTGTTGTTAGCCATAAAGTGATAACCTTTAATc                                                                                                                                                                                                                <  1:229526/301‑1 (MQ=255)`  
                                                                                                    `gggTTCCCTAAGGGTT‑GGGGATGACCCTTGGGTTTGTCTTTGGGTGTTACCTTGA‑‑GTGTCGCTCTGTGTCCCTATCTGTTACAGTCTCCTAAAGTATCCTCCTAAAGTCACCTCCTAACGGCCATCCTAAAGCCAACACCTAAAGCCTACACCTAAAGACCCACCAAGTCAACGCCTATCTTAAAGTTTAAACATAAAGACCAGACCTAAAGACCAGACCTAAAGACACTACATAAAGACCAGACCTAAAGACGCCTTGTTGTTAGCCATAAAGTGATAACCTTTAATCATTGTCTttatt                                                                                                                                                                                                    <  1:145499/301‑1 (MQ=255)`  
                                                                                                         `gagacaGGGGT‑TGGGATGACCCTTGGGTTTGTCTTTGGGTGTTACCTTGA‑‑GTGTCTCTCTGTGTCCCTATCTGTTACAGTCTCCTAAAGTATCCTCCTAAAGTCACCTCCTAACGTCCATCCTAAAGCCAACACCTAAAGCCGACACCTAAAGACCCATCAAGTCAACGCCTATCTTAAAGTTTAAACATAAAGACCAGACCTAAAGACCAGACCTAAAGACACTACATAAAGACCAGACCTAAAGACGCCTTGTTGTTAGCCATAAAGTGATAACCTTTAATCATTGTCTTTATTAATAc                                                                                                                                                                                               <  1:186272/296‑1 (MQ=255)`  
                                                                                                         `cccTAAGGGTT‑GGGGATGACCCTTGGGTTTGTCTTTGGGTGTTACCTTGA‑‑GTGTCTCTCTGTGTCCCTATCTGTTACAGTCTCCTAAAGTATCCTCCTAAAGTCACCTCCTAACTTCCATCCTAAAGCCAACACCTAAAGCCTACACCTAAAGACCCATCAAGTCAACGCCTATCTTAAAGTTTAAACATAAAGACCAGACCTAAAGACCAGACCTAAAGACACTACATAAAGACCAGACCTAAAGACGCCTTGTTGTTAGCCATAAAGTGATAAACTTTAATAATTGTCTTTATTAATaa                                                                                                                                                                                               >  1:52962/1‑300 (MQ=255)`  
                                                                                                         `cccTAAGGGTT‑GGGGATGACCCTTGGGTTTGTCTTTGGCTGTTACCTTGA‑‑GTGTCTCTCTGTCTTCATCTCTGTTACAGTCTCCTAAAGTATCCTCCTAACGTCACCTCCTAACGTCTATCCTAAAGCCAACACCTAAAGCCTACACCTACAGACCCAGCAAGTCAACGCCTATCTTTAAGTTTAAACATTAAGACCAGACCTAAAGACCAACCCTAAATACACTACATAAACACCAGACCAAATGACGCCTTGTTGTTAGCCATAAAGTGCTAACCTATAATCATTGTCTttattattat                                                                                                                                                                                               >  1:157823/1‑300 (MQ=255)`  
                                                                                                         `agacaggggTT‑GGGGATGACCCTTGGGTTTGTCTTTGGGTGTTACCTTGA‑‑GTGTCTCTCTGTGTCCCTATCTGTTACAGTCTCCTAAAGTATCCTCCTAAAGTCACCTCCTAACGTCCATCCTAAAGCCAACACCTAAAGCCTACACCTAAAGACCCATCAAGTCAACGCCTATCTTAAAGTTTAAACATAAAGACCAGACCTAAAGACCATACCTAAAGACACTACATAAAGACCAGACCTAAAGACGCCTTGTTGTTAGCCATAAAGTGATAACCTTTAATCATTGTCTTTATTAATAc                                                                                                                                                                                               <  1:227170/295‑1 (MQ=255)`  
                                                                                                               `gggTT‑GGGGATGACCCTTGGGTTTGTCTTTGGGTGTTACCTTGA‑‑GTGTCTCTCTGTGTCCCTATCTGTTACAGTCTCATAAAGTATCCTCCTAAAGTCACCTCCTAACGTCCATCCTAAAGCCAACACCTAAAGCCTACACCTAAAGACCCATCAAGTCAACGCCTATCTTAAAGTTTAAACATAAAGACCTGACCTAAAGACCAAACCTAAAGACACTACATAAAGACCAGACCTAAAGACGCCTTGTTGTTAGCCATAAAGTGATAACCTTTAATCATTGTCTttattctgtctcttat                                                                                                                                                                                         >  1:345220/1‑290 (MQ=255)`  
                                                                                                                       `ggATGACCCTTGGGTTTGTCTTTGGGTGTTACCTTGA‑‑GTGTCTCTCTGTGTCCCTATCTGTTACAGTCTCCTAAAGTATCCTCCTAAAGTCACCTCCTAACGTCCATCCTAAAGCCAACACCTAAAGCATACACCTAAAGACCCATCAAGTCAACGCCTATCTTAAAGTTTAAACATAAAGACCAGACCTAAAGACCAGACCTAAAGACACTACATAAAGACCAGACCTAAAGACGCCTTGTTGTTAGCCATAAAGTGATAAACTTTAATCATTGTCTTTATTAATACAACTCACGATAAg                                                                                                                                                                                  >  1:46655/1‑301 (MQ=255)`  
                                                                                                                         `aTGACCCTTGGGTTTGTCTTTGGGTGTTACCTTGA‑‑GTGTCTCTCTGTGTCCCTATCTGTTACAGTCTCCTAAAGTATCCTCCTAAAGTCACCTCCTAACGTCCATCCTAAAGCCAACACCTAAAGCCTACACCTAAAGACCCATCAAGTCAACGCCTATCTTAAAGTTTAAACATAAAGACCAGACCTAAAGACCAGACCTAAAGACACTACATAAAGACCAGACCTAAAGACGCCTTGTTGTTAGCCATAAAGTGATAACCTTTAATCATTGTCTTTATTAATACAACTCACTATAAGga                                                                                                                                                                                <  1:79493/301‑1 (MQ=255)`  
                                                                                                                             `cccTTGGGTTTGTCTTTGGGTGTTACCTTGA‑‑GTGTCTCTCTGTGTCCCTATCTGTTACAGTCTCCTAAAGTATCCTCCTAAAGTCACCTCCTAACGTCCATCCTAAATCCAACACCTAAAGCCTACACCTAAAGACCCATCAAGTCAACGCCTATCTTAAAGTTTAAACATAAAGACCAGACCTAAAGACCAGACCTAAAGACACTACATAAAGACCAGACCTAAAGACGCCTTGTTGTTAGCCATAAAGTGATAACCTTTAATCATTGTCTTTATTAATACAACTCACTATAAGgagaga                                                                                                                                                                            >  1:310920/1‑301 (MQ=255)`  
                                                                                                                             `cccTTGGGTTTGTCTTTGGGTGTTACCTTGA‑‑GTGTCTCTCTGTGTCCCTATCTGTTACAGTCTCCTAAAGTATCCTCCTAAAGTCACCTCCTAACGTCCATCCTAAAGCCAACACCTAAAGCCTACACCTAAAGACCCATCAAGTCAACGCCTATCTTAAAGTTTAAACATAAAGACCAGACCTAAAGACCAGACCTAAAGACACTACATAAAGACCAGACCTAAAGACGCCTTGTTGTTAGCCATAAAGTGATAAACTTTAATCATTGTCTTTATTAATACAACTCACTATAAggctgtt                                                                                                                                                                            >  1:219305/1‑296 (MQ=255)`  
                                                                                                                             `cccTTGGGTTTGTCTTTGGGTGTTACCTTGA‑‑GTGTCTCTCTGTGTCCCTATCTGTTACAGTCTCCTAAAGTATCCTCCTAAAGTCACCTCCAAACGTCCATCCTAAAGCCAACACCTAAAGCCTACACCTAAAGACCCATCAAGTCAACGCCTATCTTAAAGTTTAAACATAAAGACCAGACCTAAAGACCAGACCTAAAGACACTACATAAAGACCAGACCTAAAGACGCCTTGTTGTTAGCCATAAAGTGATAACCTTTAATCATTGTCTTTATTAATACAACTCACTATAAGgagaga                                                                                                                                                                            >  1:291226/1‑301 (MQ=255)`  
                                                                                                                                       `tGTCTTTGGGTGTTACCTTGA‑‑GTGTCTCTCTGTGTCCCTATCTGTTACAGTCTCCTAAAGTATCCTCCTAAAGTCACCTCCTAACGTCCATCCTAAAGCCAACACCTAAAGCCTACACCTAAAGACCCATCAAGTCAACGCCTATCTTAAAGTTTAAACATAAAGACCAGACCTAAAGACCAGACCTAAAGACACTACATAAAGACCAGACCTAAAGACGCCTTGTTGTTAGCCATAAAGTGATAACCTTTAATCATTGTCTTTATTAATACAACTCACTATAAGGAGAGACAACTTAAag                                                                                                                                                                  <  1:293813/301‑1 (MQ=255)`  
                                                                                                                                        `gTCTTTGGGTGTTACCTTGA‑‑GTGTCTCTCTGTGTCCCTTTCTGTTACAGTCTCCTAAAGTATCCTCCTAAAGTCACCTCCTAACGTCCATCCTAAAGCCAACAACTAAAGCCTACACCTAAAGACCCATCAAGTCAACGCCTATCTCAAAGTTTAAAAATAAAGACCAGACCTAAAGACCAGACCTAAAGACAATACATAAAGACAAGACCTAAAGACGCCTTGTTGTTAACCATAAAGTGATAACCTTTAATCATTGTCTTTATTAATAAAACTCAATCTAAGGAGAGACAAATTAAaga                                                                                                                                                                 >  1:295893/1‑301 (MQ=255)`  
                                                                                                                                        `gTCTTTGGGTGTTACCTTGA‑‑GTGTCTCTCTGTGTCCCTATCTGTTACAGTCTCCTAACGTATCCTCCTAAAGTCACCTCCTAACGTCCATCCTAAAGCCAACACCTAAAGCCTACACCTAAAGACCCATACAGTCAACGCCTATCTTAAAGTTTAAACATAAAGACCAGACCTAAAGACCAGACCTAAAGACACTACATACATACCAGACCTAAAGACGCCTTGTTTTTAGCCATAAATTGCTAACCTTTTATCTTTGTCTTTATTAATCAAACCCACtatattgtctctcatacacttat                                                                                                                                                                 >  1:224573/1‑282 (MQ=255)`  
                                                                                                                                        `gTCTTTGGGTGTTACCTTGA‑‑GTGTCTCTCTGTGTCCCGATCTGTTACAGTCTCCTAAAGTATCCTCCTAAAGTCACCTCCTAACGTCCATCCTAAAGCCAACACCTAAAGCCTACACCTAAAGACCCATCAAGTCAACGCCTATCTTAAAGTTTAAAAATAAAGACCAGACCTAAAGACCAGACCTAAAGACACTACATAAAGACCAGACCTAAAGACGCATTGTTGTTAGCCATAAAGTGATAACCTTGAATCATTGTCTTTATTAATACAAATCACTATAAGGAGAGACAACTTAAaga                                                                                                                                                                 >  1:77625/1‑301 (MQ=255)`  
                                                                                                                                         `tCTTTGGGTGTTACCTTGA‑‑GTGTCTCTCTGTGTCCCTATCTGTTACAGTCTCCTAAAGTATCCTCCTAAAGTCACCTCCTAACGTCCATCCTAAAGCCAACACCTAAAGCCTACACCTAAAGACCCATCAAGTCAACGCCTATCTTAAAGTTTAAACATAAAGACCAGACCTAAAGACCAGACCTAAAGACACTACATAAAGACCAGACCTAAAGACGCCTTGTTGTTAGCCATAAAGTGATAACCTTTAATCATTGTCTTTATTAATACAACTCACTATAAGGAGAGACAACTTAAagag                                                                                                                                                                <  1:231688/301‑1 (MQ=255)`  
                                                                                                                                                  `gtTACCTTGA‑‑GTGTCTCTCTGTGTCCCTATCTGTTACAGTCTCCTAAAGTATCCTCCTAAAGTCACCTCCTAACGTCCATCCTAAAGCCAACACCTAAAGCCTACACCTAAAGACCCATCAAGTCAACGCCTATCTTAAAGTTTAAACATAAAGACCAGACCTAAAGACCAGACCTAAAGACACTACATAAAGACCAGACCTAAAGACGCCTTGTTGTTAGCCATAAAGTGATAACCTTTAATCATTGTCTTTATTAATACAAATCACTATAAGGAGAGACAACTTAAAGAGACTTAAAAg                                                                                                                                                       >  1:26295/1‑301 (MQ=255)`  
                                                                                                                                                    `tACCTTGA‑‑GTGTCTCTCTGTGTCCCTATCTGTTACAGTCTCCTAAAGTATCCTCCTAAAGTCACCTCCTAACGTCCATCCTAAAGCCAACACCTAAAGCCTACACCTAAAGACCCATCAAGTCAACGCCTATCTTAACGTTTAAACATAAAGACCAGACCTAAAGACCAGACCTAAAGACACTACATCAAGACCAGACCTAAAGCCGCCTTGTTGTTAGCCATAACGTGATCACCTTTAATCATTGTCTTTATTAATACAACTCACTATACGGAGAGACAACTTAAAGAGACTTACAAGAt                                                                                                                                                     >  1:65823/1‑301 (MQ=255)`  
                                                                                                                                                    `tACCTTGA‑‑GTGTCTCTCTGTGTCCCTATCTGTTACAGTCTCCTAAAGTATCCTCCTAAAGTCACCTCCTAACGTCCATCCTAAAGCCAACACCTAAAGCCTACACCTAAAGACCCATCAAGTCAACGCCTATCTTAAAGTTTAAACATAAAGACCAGACCTAAAGACCAGACCTAAAGACACTACATAAAGACCAGACCTAAAGACGCCTTGTTGTTAGCCATAAAGTGATAACCTTTAATAATTGTCTTTATTAATACAACTCACTATAAGGAGAGACAACTTAAAGAGACTTAAAAGAt                                                                                                                                                     >  1:256951/1‑301 (MQ=255)`  
                                                                                                                                                          `gA‑‑GTGTCTCTCTGTGTCCCTATCTGTTACAGTCTCCTAAAGTATCCTCCTAAAGTCACCTCCTAACGTCCATCCTAAAGCCAACACCTAAAGCCTACACCTAAAGACCCATCAAGTCAACGCCTATCTTAAAGTTTAAACATAAAGACCAGACCTAAAGACCAGACCTAAAGACACTACATAAAGACCAGACCTAAAGACGCCTTGTTGTTAGCCATAAAGTGATAACCTTTAATCATTGTCTTTATTAATACAACTCACTATAAGGAGAGACAACTTAAAGAGACTTAAAAGATTAAttt                                                                                                                                               >  1:6887/1‑301 (MQ=255)`  
                                                                                                                                                                 `aacagctatgtCCTTATCTGTTACAGTCTCCTAAAGTATCCTCCTAAAGTCACCTCCTAACGTCCATCCTAAAGCCAACACCTAAAGCCTACACCTAAAGACCCATCAAGTCAACGCCTATCTTAAAGTTTAAACATAAAGACCAGACCTAAAGACCAGACCTAAAGACACTACATAAAGACCAGACCTAAAGACGCCTTGTTGTTAGCCATAAAGTGATAACCTTTAATCATTGTCTTTATTAATACAACTCACTATAAGGAGAGACAACTTAAAGAGACTTAAAAGATTAATTTAAAAt                                                                                                                                          <  1:328558/293‑1 (MQ=255)`  
                                                                                                                                                                    `ctctGTGTCCCTATCTGTTACAGTCTCCTAAAGTATCCTCCTAAAGTCACCTCCTAACGTCCATCCTAAAGCCAACACCTAAAGCCTACACCTAAAGACCCATCAAGTCAACGCCTATCTTAAAGTTTAAACATAAAGACCAGACCTAAAGACCAGACCTAAAGACACTACATAAAGACCAGACCTAAAGACGCCTTGTTGTTAGCCATAAAGTGATAACCTTTAATCATTGTCTTTATTAATACAACTCACTATAAGGAGAGACAACTTAAAGAGACTTAAAAGATTAATTTAAAATTTa                                                                                                                                       >  1:269562/1‑301 (MQ=255)`  
                                                                                                                                                                            `cccTATCTGTTACAGTCTCCTAAAGTATCCTCCTAAAGTCACCTCCTAACGTCCATCCTAAAGCCAACACCTAAAGCCTACACCTAAAGACCCATCAAGTCAACGCCTATCTTAAAGTTTAAACATAAAGACCAGACCTAAAGACCAGACCTAAAGACACTACATAAAGACCAGACGTAAAGACGCCTTGTTGTTAGCCATAAAGTGATAACCTTTAATCATTGTCTTTATTAATACAACTCACTATAAGGAGAGACAACTTAAAGAGCCTTAAAAGCTTAATTTAAAATTTATCaaaaaa                                                                                                                               >  1:100046/1‑300 (MQ=255)`  
                                                                                                                                                                                `aTCTGTTACAGTCTCCTAAAGTATCCTCCTAAAGTCACCTCCTAACGTCCATCCTAAAGCCAACACCTAAAGCCTACACCTAAAGACCCATCAAGTCAACGCCTATCTTAAAGTTTAAACATAAAGACCAGACCTAAAGACCAGACCTAAAGACACTACATAAAGACCAGACCTAAAGACGCCTTGTTGTTAGCCATAAAGTGATAACCTTTAATCATTGTCTTTATTAATACAACTCACTATAAGGAGAGACAACTTAAAGAGACTTAAAAGATTAATTTAAAATTTATCAAAAAGAGTa                                                                                                                           >  1:298588/1‑301 (MQ=255)`  
                                                                                                                                                                                `aTCTGTTACAGTCTCCTAAAGTATCCTCCTAAAGTCACCTCCTAACGTCCATCCTAAAGCCAACACCTAAAGCCTACACCTAAAGACCCATCAAGTCAACGCCTATCTTAAAGTTTAAACATAAAGACCAGACCTAAAGACCAGACCTAAAGACACAACATAAAGACCAGACCTAAAGACGCCTTGTTGTTAGCCATAAAGTGATAACCTTTAATCATTGTCTTTATTAATACAACTCACTATAAGGAGAGACAACTTAAAGAGACTTAAAAGATTAATTTAAAATTTATCAAAAAGAGTa                                                                                                                           >  1:11895/1‑301 (MQ=255)`  
                                                                                                                                                                                 `ataagagacagctcCTAAAGTATCCTCCTAAAGTCACCTCCTAACGTCCATCCTAAAGCCAACACCTAAAGCCTACACCTAAAGACCCATCAAGTCAACGCCTATCTTAAAGTTTAAACATAAAGACCAGACCTAAAGACCAGACCTAAAGACACTACATAAAGACCAGACCTAAAGACGCCTTGTTGTTAGCCATAAAGTGATATCCTTTAATCATTGTCTTTATTAATACAACTCACTATAAGGAGAGACAACTTAAAGAGACTTAAAAGATTAATTTAAAATTTATCAAAAAGAGTAt                                                                                                                          <  1:307583/290‑1 (MQ=255)`  
                                                                                                                                                                                  `cTGTTACAGTCTCCTAAAGTATCCTCCTAAAGTCACCTCCTAACGTCCATCCTAAAGCCAACACCTAAAGCCTACACCTAAAGACCCATCAAGTCAACGCCTATCTTAAAGTTTAAACATAAAGACCAGACCTAAAGACCAGACCTAAAGACACTACATAAAGACCAGACCTAAAGACGCCTTGTTGTTAGCCATAAAGTGATAACCTTTAATCATTGTCTTTATTAATACAACTCACTATAAGGAGAGACAACTTAAAGAGACTTAAAAGATTAATTTAAAATTTATCAAAAAGAGTAtt                                                                                                                         >  1:110860/1‑301 (MQ=255)`  
                                                                                                                                                                                     `gagacagctcCTAAAGTATCCTCCTAAAGTCACCTCCTAACGTCCAGCCTAAAGCCAACACCTAAAGCCTACACCTAAAGACCCATCAAGTCAACGCCTATCTTAAAGTTTAAACATAAAGACCAGACCTAAAGACCAGACCTAAAGACACTACATAAAGACCAGACCTAAAGACGCCTTGTTGTTAGCCATAAAGTGATAACCTTTAATCATTGTCTTTATTAATACAACTCACTATAAGGAGAGACAACTTAAAGAGACTTAAAAGATTAATTTAAAATTTATCAAAAAGAGTATTGAc                                                                                                                      <  1:174141/294‑1 (MQ=255)`  
                                                                                                                                                                                            `ctcCTAAAGTATCCTCCTAAAGTCACCTCCTAACGTCCATCCTAAAGCCAACACCTAAAGCCTACACCTAAAGACCCATCAAGTCAACGCCTATCTTAAAGTTTAAACATAAAGACCAGACCTAAAGACCAGATCTAAAGACACTACATAAAGACCAGACCTAAAGACGCATTGTTGTTAGCCATAAAGTGATAACATTTAATCATTATCTTTATTAATACAACTCACTATAAGGAGAGACAACTTAAAGAGACTTAAACGATTAATTTAACATTTATCAAAAAGAGTATTGATTTAAAGt                                                                                                               >  1:134201/1‑301 (MQ=255)`  
                                                                                                                                                                                            `ctcCTAAAGTATCCTCCTAAAGTCACCTCCTAACGTCCATCCTAAAGCCAACACCTAAAGCCTACACCTAAAGACCCATCAAGTCAACGCCTATCTTAAAGTTTAAACATAAAGACCAGACCTAAAGACCAGACTTAAAGACACTACATAAAGACAAGACCTAACGACGCCTTGTTGTTAGCCATAAAGTGATAACCTTTAATCTTTGTCTTTATTACTACAACTCACTATACGTAGAGACACCTTAAAGAGACTTAAAAGATTAATTTAAAATTTATCAAAAAGAGTATTGACTAAAAGt                                                                                                               >  1:238698/1‑301 (MQ=255)`  
                                                                                                                                                                                            `ctcCTAAAGTATCCTCCTAAAGTCACCTCCTAACGTCCATCCTAAAGCCAACACCTAAAGCCTACACCTAAAGACCCATCAAGTCAACGCCTATCTTAAAGTTTAAACATAAAGACCAGACCTAAAGACCAGACCTAAAGACACTACATAAAGACCAGACCTAAAGACGCCTTGTTGTTAGCCATAAAGTGATAACCTTTAATCATTGTCTTTATTAATACAACTCACTATAAGGAGAGACAACTTAAAGAGACTTACAAGATTAATTTACAATTTATCAAAAAGAGTAtctgtctcttat                                                                                                               >  1:121682/1‑290 (MQ=255)`  
                                                                                                                                                                                            `ctcCTAAAGTATCCTCCTAAAGTCACCTCCTAACGTCCATCCTAAAGCCAACACCTAAAGCCTACACCTAAAGACCCATCAAGTCAACGCCTATCTTAAAGTTTAAACATAAAGACCAGACCTAAAGACCAGACCTAAAGACACTACATAAAGACCAGACCTAAAGACGCCTTGTTGTTAGCCATAAAGTGATAACCTTTAATCATTGTCTTTATTAATACAACTCACTATAAGGAGAGACAACTTAAAGAGACTTAAAAAATTAATTTAAAATTTATCAAAAAGAGTATTGACTaaaaaa                                                                                                               >  1:93856/1‑299 (MQ=255)`  
                                                                                                                                                                                                 `aaaGTATCCTCCTAAAGTCACCTCCTAACGTCCATCCTAAAGCCAACACCTAAAGCATACACCTAAAGACCCATCAAGTCAACGCCTATCTTAAAGTTTAAACATAAAGACCAGACCTAAAGACCAGACCTAAAGACACTACATAAAGACCAGAACTAAAGACGCCTTGTTGTTAGCCATAAAGTGATAACCTTTAATCATTGTCTTTATTAATACAACTCACTATAAGGTGAGACAACTTAAAGAGACTTAAAAGATTAATTTAAAATTTATCAAAAAGAGTATTGACTTAAAGTCTAAc                                                                                                          >  1:136545/1‑301 (MQ=255)`  
                                                                                                                                                                                                  `aagacagctcctAAAGTCACCTCCTAACGTCCATCCTAAAGCCAACACCTAAAGCCTACACCTAAAGACCCATCAAGTCAACGCCTAACTTAAAGTTTAAACATAAAGACCAGACCTAAAGACCAGACCTAAAGACACTACATAAAGACCAGACCTAAAGACGCCTTGTTGTTAGCCATAAAGTGATAACCTTTAATCATTGTCTTTATTAATACAACTCACTATAAGGAGAGACAACTTAAAGAGACTTAAAAGATTAATTTAAAATTTATCAAAAAGAGTATTGACTTAAAGTCTAAcc                                                                                                         <  1:245618/294‑1 (MQ=255)`  
                                                                                                                                                                                                       `aagacagaaaGTCACCTCCTAACGTCCATCCTAAAGCCAACACCTAAAGCCTACACCTAAAGACCCCTCAAGTCAACGCCTATCTTAAAGTTTAAACATAAAGACCAGACCTAAAGACCAGACCTAAAGACACTACATAAAGACCAGACCTAAAGACGCCTTGTTGTTAGCCATAAAGTGATAACCTTTAATCATTGTCTTTATTAATACAACTCACTATAAGGAGAGACAACTTAAAGAGACTTAAAAGATTAATTTAAAATTTATCAAAAAGAGTATTGACTTAAAGTCTAACCTATAg                                                                                                    <  1:6209/294‑1 (MQ=255)`  
                                                                                                                                                                                                           `cctAAAGTCACCTCCTAACGTCCATCCTAAAGCCAACACCTAAAGCCTACACCTAAAGACCCATCAAGTCAACGCCTATCTTAAAGTTTAAACATAAAGACCAGACCTAAAGACCAGACCTAAAGACACTACATAAAGACCAGACCTAAAGACGCCTTGTTGTTAGCCATAAAGTGATAACCTTTAATCATTGTCTTTATTAATACAACTCACTATAAGGAGAGACAACTTAAAGAGACTTAAAAGATTAATTTAAAATTTATCAAAACGAGTATTGACTTAAAGTCTAACCTATAGGATa                                                                                                >  1:317397/1‑301 (MQ=255)`  
                                                                                                                                                                                                               `aaGTCACCTCCTAACGTCCATCCTAAAGCCAACACCTAAAGCCTACACCTAAAGACCCATCAAGTCAACGCCTATCTTAAAGTTTAAACATAAAGACCAGACCTAA‑‑‑‑‑AGACCTAAAGACACTACATAAAGACCAGACCTAAAGACGCCTTGTTGTTAGCCATAAAGTGATAACCTTTAATCATTGTCTTTATTAATACAACTCACTATAAGGAGAGACAACTTAAAGAGACTTAAAAGATTAATTTAAAATTTATCAAAAAGAGTATTCACTTAAAGTCTAACCTATAGGATACTTACAGcc                                                                                       <  1:108352/301‑1 (MQ=255)`  
                                                                                                                                                                                                                   `cACCTCCTAACGTCCATCCTAAAGCCAACACCTCAACCCTACACCTAAAGACCCCTCAAGTCAACGCCTATCTTAAAGTTTAAACATAAAGACCCGACCTAAAGACCAGACCTAAAGACACTACATAAAGACCAGACCTAAAGACGCCTTGTTGTTAGCCATAAAGTGATAACCTTTAATCATTGTCTTTATTAATACAACTCACTATAAGGAGAGACAACTTAAAGAGACTTAAAAGATTAATTTAAAATTTATCAAAAAGAGTATTGACTTAAAGTCTAACCTATAGGATACTTACAGc                                                                                        <  1:124611/301‑1 (MQ=255)`  
                                                                                                                                                                                                                    `gcctcctAACGTCCATCCTAAAGCCAACACCCAAAGCCTACACCTAAAGACCCATCAAGTCAACGCCTATCTTAAAGTTTAAACATAAAGACCAGACCTAAAGACCAGACCTAAAGACACTACATAAAGACCAGACCTAAAGACGCCTTGTTGTTAGACATAAAGTGATAACCTTTAATCATTGTCTTTATTAATACAACTCACTATAAGGAGAGACAACTTAAAGAGACTTAAAAGATTAATTTAAAATTTATCAAAAAGAGTATTGACTTAAAGTCTAACCTATAGGATACTTACAGcc                                                                                       <  1:195445/300‑1 (MQ=255)`  
                                                                                                                                                                                                                     `cctcctAACGTCCATCCTAAAGCCAACACCTAAAGCCTACACCTAAAGACCCATCAAGTCAACGCATATCTTAAAGTTTAAACATAAAGACCAGACCTAAAGACCAGACCTAAAGACACTACATAACGACCAGACCTAAAGACGCCTTGTTATTAGCCATAAAGTGAAAACCTTTAATCATTGTCTTTATTAATACAACTCACTATAAGGAGAGACAACTTAAAGAGCCTTCACAGATTAATTTAAAATTTATCAAACAGAGTATTGACTTAAAGTCTAACATATAGGATACTTACAGCCa                                                                                      >  1:171294/1‑301 (MQ=255)`  
                                                                                                                                                                                                                     `cctcctAACGTCCATCCTAAAGCCAACACCTAAAGCCTACACCTAAAGACCCATCAAGTCAACACCTATCTTAAAGTTTAAACATAAAGACCAGACCTAAAGACCAGACCTAAAGACACTACATAAAGACCAGACCTAAAGACGCCTTGTTGTTAGCCATAAAGTGATAACCTTTAATCATTGTCTTTATTAATACAACTCACTATAAGGCGATACAACTTAAAGAGACTTAAAAGATTAATTTAAAATTTATCAAAAAGAGTATTGACTTAACGTCTAACCTCTATGATACTTACAGCCa                                                                                      >  1:252055/1‑301 (MQ=255)`  
                                                                                                                                                                                                                      `ctcctAACGTCCATCCTAAAGCCCACACCTAAAGCCTACACCTAAAGACCCATCAAGTCAACGCCTATCTTAAAGTTTAAACATAAAGACCAGACCTAAAGACCAGACCTAAAGACACTACATAAAGACCAGACCTAAAGACGCCTTGTTGTTAGCCATAAAGTGATAACCTTTAATCATTGTCTTTATTAATACAACTCACTATAAGGAGAGACAACTTAAAGAGACTTAAAAGATTAATTTAAAATTTATCAAAAAGAGTATTGACTTAAAGTCTAACCTATAGGATACTTACAGCCAt                                                                                     <  1:208145/301‑1 (MQ=255)`  
                                                                                                                                                                                                                      `ctcctAACGTCCATCCTAAAGCCAACACCTAAAGCCTCCACCTCAAGACCCATCAAGTCAACGCCTATCTTAAAGTTTAAACATAAAGACCAGACCTAAAGACCAGACCTAAAGACACTACATAAAGACCAGACCCCAAGCCGCCTTGTTGTTAGCCATAAAGTGATAACCTTTAATCATTGTCTTTATTAATACAACTCACTATACGGAGAGACAACTTAAAGAGACTTAAAAGATTAATTTAAAATTTATCAAAAAGAGTATTGACTTAAAGTCTAACCTATAGGATACTTCCAGcccc                                                                                     >  1:75256/1‑299 (MQ=255)`  
                                                                                                                                                                                                                          `tAACGTCCATCCTAAAGCCAACACCTAAAGCCTACACCTAAAGACCCATCAAGTCAACGCCTATCGTAAAGTTTAAACATAAAGACCAGACCTAAAGACCAGACCTAAAGACACTACATAAAGACCAGACCTAAAGACGCCTTGTTGTTAGCCATAAAGTGATAACCTTTAATCATTGTCTTTATTAATACAACTCACTATAAGGAGAGACAACTTAAAGAGACTTAAAAGATTAATTTAAAATTTATCAAAAAGAGTATTGACTTAAAGTCTAACCTATAGGATACTTACAGACATCgag                                                                                 <  1:320228/301‑1 (MQ=255)`  
                                                                                                                                                                                                                             `cGTCCATCCTAAAGCCAAAACCTAAAGCCTACACCTAAAGACCCATCAAGTCAACGCCTATCTTAAAGTTTAAACATAAAGACCAGACCTAAAGACCAGACCTAAAGACACTACATAAAGACCAGACCTAAAGACGCCTTGTTGTTAGCCATAAAGTGATAACCTTTAATCATTGTCTTTATTAATACAACTCACTATAAGGAGAGACAACTTAAAGAGACTTAAAAGATTAATTTAAAATTTATCAAAAAGAGTATTGACTTAAAGTCTAACCTATAGGATACTTACAGCCATCGAGAgg                                                                              <  1:8637/301‑1 (MQ=255)`  
                                                                                                                                                                                                                             `agacagtCCTAAAGCCAACACCTAAAGCCTACCCCTAAAGAACCATCAAGTCAACGCCTATCTTAAAGTTTAAACATAAAGACCAGACCTAAAGACCAGACCTAAAGACACTACATAAAGACCAGACCTAAAGACGCCTTGTTGTTAGCCATAAAGTGATAACCTTTAATCATTGTCTTTATTAATACAACTCACTATAAGGAGAGACAACTTAAAGAGACTTAAAAGATTAATTTAAAATTTATCAAAAAGAGTATTGACTTAAAGTCTAACCTATAGGATACTTACAGCCATCGAGAgg                                                                              <  1:249141/295‑1 (MQ=255)`  
                                                                                                                                                                                                                                    `ccTAAAGCCAACACCTAAAGCCTACACCTAAAGACCCATCAAGTCAACGCCTATCTTAAAGTTTAAACATAAAGACCAGACCTAAAGACCAGACCTAAAGACACTACATAAAGACCAGACCTAAAGACGCCTTGTTGTTAGCCATAAAGTGATAACCTTTAATCATTGTCTTTATTAATACAACTCACTATAAGGAGAGACAACTTAAAGAGACTTAAAAGATTAATTTAAAATTTATCAAAAAGAGTATTGACTTAAAGTCTAACCTATAGGATACTTACAGCCATCGAGAGGGACACgg                                                                       >  1:284640/1‑301 (MQ=255)`  
                                                                                                                                                                                                                                     `cTAAAGCCAACACCTAAAGCCTACACCTAAAGACCCATCAAGTCAACGCCTATCTTAAAGTTTAAACATAAATACCAGACCTAAAGACCAGACCTAAAGACACTACATAAAGACCAGACCTAAAGACGCCTTGTTGTTAGCCATAAAGTGATAACCTTTAATCATTGTCTTTATTAATACAACTCACTATAAGGAGAGACAACTTAAAGAGACTTAAAAGATTAATTTAAAATTTATCAAAAAGAGTATTGACTTAAAGTCTAACCTATAGGATACTTACAGCCATCGAGAGGGACACGGc                                                                      <  1:103547/301‑1 (MQ=255)`  
                                                                                                                                                                                                                                               `cacCTAAAGCCTACACCTAAAGACCCATCAAGTCAACGCCTATCTTAAAGTTTAAACATAAAGACCAGACCTAAAGACCAGACCTAAAGACACTACATAAAGACCAGACCTAAAGACGCCTTGTTGTTAGCCATAAAGTGATAACCTTTAATCATTGTCTTTATTAATACAACTCACTATAAGGAGAGACAACTTAAAGAGACTTAAAAGATTAATTTAAAATTTATCAAAAAGAGTATTGACTTAAAGTCTAACCTATAGGATACTTACAGCCATCTAGAGGGACACGGCCAATAGCCAt                                                            >  1:39037/1‑301 (MQ=255)`  
                                                                                                                                                                                                                                               `cacCTAAAGCCTACACCTAAAGACCCATCAAGTCAACGCCTATCTTAAAGTTTAAACATAAAGACCAGACCTAAAGACCAGACCTAAAGACACTACATAAAGACCAGACCTAAAGACGCCTTGTTGTTAGCCATAAAGTGATAACCTTTAATCATTGTCTTTATTAATACAACTCACTATAAGGAGAGACAACTTAAAGAGACTTAAAAGATTAATTTAAAATTTATCAAAAAGAGTATTGACTTAAAGTCTAACCTATAGGATACTTAAAGCCATCGAGAGGGACACGGCGAATAGCCaa                                                            >  1:55469/1‑300 (MQ=255)`  
                                                                                                                                                                                                                                                 `ccTAAAGCCTACACCTAAAGACCCATCAAGTCAACGCCTATCTTAAAGTTTAAACATAAAGACCAGACCTAAAGACCAGACCTAAAGACACTACATAAAGACCAGACCTAAAGACGCCTTGTTGTTAGCAATAAAGTGATAACCTTTAATCATTGTATTTATTAATACAACTCACTATAAGGAGAGACAACTTAAAGAGACTTAAAAGATTAATTTAAAATTTATCAAAAAGAGTATTGACTTAAAGTCTAACCTATACAATACTTACAGCCATCGAAAGGGACACGGCAAATAGCCATcc                                                          >  1:189330/1‑301 (MQ=255)`  
                                                                                                                                                                                                                                                       `gCCTACACCTAAAGACCCATCAAGTCAACGCCTATCTTAAAGTTTAAACATAAAGACCAGACCTAAAGACCAGACCTAAAGACACTACATAAAGACCAGACCTAAAGACGCCTTGTTGTTAGCCATAAAGTGATAACCTTTAATCATTGTCTTTATTAATACAACTCACTATAAGGAGAGACAACTTAAAGAGACTTAAAAGATTAATTTAAAATTTATCAAAAAGAGTATTGACTTAAAGTCTAACCTATAGGATACTTACAGCCATCGAGAGGGACACGGCGAATAGCCATCCCAATCg                                                    <  1:116487/301‑1 (MQ=255)`  
                                                                                                                                                                                                                                                         `cTACACCTAAAGACCCATCAAGTCAACGCCTATCTTAAAGTTTAAACATAAAGACCAGACCTAAAGACCAGACCTAAAGACACTACATAAAGACCAGACCTAAAGACGCCTTGTTGTTAGCCATAAAGTGATAACCTTTAATCATTGTCTTTATTAATACAACCCACTATAAGGAGAGACAACTTAAAGAGACTTAAAAGATTAATTTAAAATTTATCAAAAAGAGTATTGACTTAAAGTCTAACCTATAGGATACTTACAGCCATCGAGAGGGACACGGCGAATAGCCATCCCAATCGac                                                  <  1:140204/301‑1 (MQ=255)`  
                                                                                                                                                                                                                                                              `ccTAAAGACCCATCAAGTCAACGCCTATCTTAAAGTTTAAACATAAAGACCAGACCTAAAGACCAGACCTAAAGACACTACATAAAGACCAGACCTAAAGACGCCTTGTTGTTAGCCATAAAGTGATAACCTTTAATCATTGTCTTTATTAATACAACTCACTATAAGGAGAGACAACTTAAAGAGACTTAAAAGATTAATTTAAAATTTATCAAAAAGAGTATTGACTTAAAGTCTAACCTATAGGATACTTACAGCCATCGAGAGGGACACGGCGAATAGCCATCCCAATCGCCACCgg                                             >  1:242173/1‑301 (MQ=255)`  
                                                                                                                                                                                                                                                                                `taagagacagcTTAAAGTTTAAACATAAAGACCAGACCTAAAGACCAGACCTAAAGACACTACATAAAGACCAGACCTAAAGACGCCTTGTTGTTAGCCATAAAGTGATAACCTTTAATCATTGTCTTTATTAATACAACTCACTATAAGGAGAGACAACTTAAAGAGACTTAAAAGATTAATTTAAAATTTATCAAAAAGAGTATTGACTTAAAGTCTAACCTATAGGATACTTACAGCCATCGAGAGGGACACGGCGAATAGCCATCCCAATCGACACCGGGGTCAACCGGATAAGTAg                           <  1:49461/291‑1 (MQ=255)`  
                                                                                                                                                                                                                                                                                `cAACGCCTAACTTAAAGTTTAAACATAAAGACCAGACCTAAAGACCAGACCTAAAGACACTACATAAAGACCAGACCTAAAGACGCCTTGTTGTTAGCCATAAAGTGATAACCTTTAATCATTGTCTTTATTAATACAACTCACTATAAGGAGAGACAACTTAAAGAGACTTAAAAGATTAATTTAAAATTTATCAAAAAGAGTATTGACTTAAAGTCTAACCTACAGGATACTTACAGCCATCGAGAGGGACACGGCGAATAGCCATCCCAATCGACACCGGGGTCAACCGGATAAGTAg                           <  1:113918/301‑1 (MQ=255)`  
                                                                                                                                                                                                                                                                                  `aCGCCTATCTTAAAGTTTAAACATAAAGACCAGACCTAAAGACCAGACCTAAAGACACTACATACAGACCAGACCTAAAGACGCCTTGTTGTTAGCCATAAAGTGATAACCTTTAATCATTGTCTTTATTAATACAACTCACTATAAGGAGAGACAACTTAAAGAGACTTAAAAGATTAATTTAAAATTTATCAAAAAGAGTATTGACTTAAAGTCTAACCTATAGGATACTTACATCCATCGAGAGGGACACGGCGAATAGCCATCCCAATCGACACCGGGGTCAACCGGATAAGTAGAc                         <  1:197570/301‑1 (MQ=255)`  
                                                                                                                                                                                                                                                                                  `aCGCCTATCTTAAAGTTTAAACATAAAGACCAGACCTAAAGACCAGACCTAAAGACACTACATAAAGACCAGACCTAAAGACGCCTTGTTGTTAGACATAAAGTGATAACCTTTAATCATTGTCTTTATTAATAAAACTCACTATAAGGAGAGACAACTTAAAGAGACTTAAAAGATTAATGTAAAATTTATCAAAAAGAGTATTGACTTAAAGTCTAACCTATAGGATACTTACAGCCATCGAGAGGGACACGGCGAATAGCCATCCCAATCGACACCGGGGTCAACCGGATAAGTAGAc                         <  1:234749/301‑1 (MQ=255)`  
                                                                                                                                                                                                                                                                                  `aCGACTAACGTAAAGGTTAAACAGAAAAGACAGACCTAAAGACCAGAACTAAAGACACGACATAAAGACATGACATAAAGACGACTTGTTGTTAGCAATAAAGTGATAACCTTTAATCATTGTCTTTATTAATACAACTCACTATAAAGAGAGACAACTTAAAGAGACTTAAAAGATTAATTTAAAATTTATCAAAAAGAGTATTGACTTAAAGTCTAACCTATAGGATACTTACAGCCATCGAGAGGGACACGGCGAATAGCCATCCCAATCGACACCGGGGTCAACCGGATAAGTAGAc                         <  1:82881/301‑1 (MQ=255)`  
                                                                                                                                                                                                                                                                                        `aTCTTAAAGTTTACACATAAAGACCAGACCTAAAGACCAGACCTAAAGACACTACATAAAGACCAGACCTATAGACGCCTTGTTGTTAGCCATAAAGTGATAACCTTTAATCATTGTCTTTATTAATACAACTCACTATAAGGAGAGACAACTTAAAGAGACTTAAAAGATTAATTTAAAATTTATTAAAAAGAGTATTGACTTAAAGTCTAACATATAGGATACTTACAGCCATCCAGAGGGAAACGGCGAATAGCCATCCCAATCGTCCCCGGGTCCAACCGGATAAGTatctctctct                   >  1:183550/1‑292 (MQ=255)`  
                                                                                                                                                                                                                                                                                        `aTCTTAAAGTTTAAACATAAAGACCAGACCTAAAGACCAGACCTAAAGACACTACATAAAGACCAGGCCTAAAGACGCCTTGTTGTTAGCCATAAAGTGATAACCTTTAATCATTGTCTTTATTAATACAACTCACTATAAGGAGAGACAACTTAAAGAGACTTAAAAGATTAATTTAAAATTTATCAAAAAGAGTATTGACTTAAAGTCTAACCTATAGGATACTTACAGCCATCGAGAGGGACACGGCGAATAGCCATCCCAATCGACACCGGGGTCAACCGGATAAGTAGACAGCCTg                   >  1:5947/1‑301 (MQ=255)`  
                                                                                                                                                                                                                                                                                             `aaaGTTTAAACATAAAGACCAGACCTAAAGACCAGACCTAAAGACACTACATAAAGACCAGACCTAAAGACGCCTTGTTGTTAGCCATAAAGTGATAACCTTTAATCATTGTCTTTATTAATACAACTCACTATAAGGAGAGACAACTTAAAGAGACTTAAAAGATTAATTTAAAATTTATCAAAAAGAGTATTGACTTAAAGTCTAACCTATAGGATACTTACAGCCATCGAGAGGGACACGGCGAATAGCCATCCCAATCGACACCGGGGTCAACCGGATAAGTAGACAGCCTGATAAg              <  1:193326/301‑1 (MQ=255)`  
                                                                                                                                                                                                                                                                                               `aGTTTAAACATAAAGACCAGACCTAAAGACCAGACCTAAAGACACTACATAAAGACCAGACCTAAAGACGCCTTGTTGTTAGCCATAAAGTGATAACCTTTAATCATTGTCTTTATTAATACAACTCACTATAAGGAGAGACAACTTAAAGAGACTTAAAAGATTAATTTAAAATTTATCAAAAAGAGTATTGACTTAAAGTCTAACCTATAGGATACTTACAGCCATCGAGAGGGACACGGCGAATAGCCATCCCAATCGACACCGGGGTCCACCGGATAAGTAGACAGCCTGATAAGTc            >  1:212196/1‑301 (MQ=255)`  
                                                                                                                                                                                                                                                                                                       `cATAAAGACCAGACCTAAAGACCAGACCTAAAGACACTACATAAAGACCAGACCTAAAGACGCCTTGTTGTTAGCCATAAAGTGATAACCTTTAATCATTGTCTTTATTAATACAACTCACTATAAGGAGAGACAACTTAAAGAGACTTAAAAGATTAATTTAAAATTTATCAAAAAGAGTATTGACTTAAAGTCTAACCTATAGGATACTTACAGCCATCGAGAGGGACACGGCGAATAGCCATCCCAATCTACACCGGGGTCAACCGGATAAGTAGAcctgtctcttatacacatctcc    >  1:176926/1‑280 (MQ=255)`  

                                                                                                                                                                                                                                                                                                         `|`                                                                                                                                                                                                                                                                                                              
`‑‑‑‑‑‑‑‑‑‑‑‑TCTCACAGTGTACGGACCTAAAGTTCCCCCATAGGGGGTACCTAAAG‑CCCAGCCAATCACCTAAAGTCAACCTTCGGTTGACCTTGAGGGTTCCCTAAGGGTT‑GGGGATGACCCTTGGGTTTGTCTTTGGGTGTTACCTTGA‑‑GTGTCTCTCTGTGTCCCTATCTGTTACAGTCTCCTAAAGTATCCTCCTAAAGTCACCTCCTAACGTCCATCCTAAAGCCAACACCTAAAGCCTACACCTAAAGACCCATCAAGTCAACGCCTATCTTAAAGTTTAAACATAAAGACCAGACCTAAAGACCAGACCTAAAGACACTACATAAAGACCAGACCTAAAGACGCCTTGTTGTTAGCCATAAAGTGATAACCTTTAATCATTGTCTTTATTAATACAACTCACTATAAGGAGAGACAACTTAAAGAGACTTAAAAGATTAATTTAAAATTTATCAAAAAGAGTATTGACTTAAAGTCTAACCTATAGGATACTTACAGCCATCGAGAGGGACACGGCGAATAGCCATCCCAATCGACACCGGGGTCAACCGGATAAGTAGACAGCCTGATAAGTCGCACGAAAAA  >  NC_001604.1/1‑582`

  

Alignment Legend

Aligned base mismatch/match (shaded by quality score): `ATCG/ATCG` `< 3 ≤` `ATCG/ATCG` `< 11 ≤` `ATCG/ATCG` `< 22 ≤` `ATCG/ATCG` `< 34 ≤` `ATCG/ATCG` `< 38 ≤` `ATCG/ATCG`

Unaligned base: `atcg`    Masked matching base: `atcg`    Alignment gap: `‑`    Deleted base: `‑`

Reads not counted as support for junction

read\_name Not counted due to insufficient overlap past the breakpoint.

read\_name Not counted due to not crossing MOB target site duplication.
